# MicroRNAs involve in bicuspid aortic aneurysm: pathogenesis and biomarkers

**DOI:** 10.1186/s13019-021-01613-9

**Published:** 2021-08-12

**Authors:** Hao Jia, Le Kang, Zhen Ma, Shuyang Lu, Ben Huang, Chunsheng Wang, Yunzeng Zou, Yongxin Sun

**Affiliations:** 1 Department of Cardiac Surgery, Zhongshan Hospital, Fudan University, 1069 Xietu Road, 200032 Shanghai, People’s Republic of China; 2 Central Laboratory of Cardiovascular Institute, Zhongshan Hospital, Fudan University, 1069 Xietu Road, 200032 Shanghai, People’s Republic of China

**Keywords:** miRNA, Aneurysm, Bicuspid aortic valve, Mechanism, Biomarker

## Abstract

The incidence of bicuspid aortic valves (BAV) is high in the whole population, BAV-related thoracic aortic aneurysm (TAA) is accompanied by many adverse vascular events. So far, there are two key points in dealing with BAV-related TAA. First is fully understanding on its pathogenesis. Second is optimizing surgical intervention time. This review aims to illustrate the potential role of miRNAs in both aspects, that is, how miRNAs are involved in the occurrence and progression of BAV-related TAA, and the feasibilities of miRNAs as biomarkers.

## Highlights

1. MiRNA involved in highly heterogeneous etiology of BAV-related TAA.
2. In BAV-related TAA, miRNA mainly targets ECM, VSMC and ECs.
3. MiRNA can be used as a biomarker to guide the timing of TAA surgery.

## Introduction

Bicuspid aortic valve (BAV) is one of the most prevalent congenital heart malformation with overall incidence of 1% to 2% [1]. This malformation is closely associated with a high risk of aortic valve dysfunction and thoracic aortic complications. In patients with BAV, the progressive dilation of thoracic aorta will be a critical focus complication. Without effective surgical intervention, progressive aortic dilation can result in aortic dissection (mortality of which averages 25% in its acute Stanford A-type) [2] and aortic rupture (mortality of which averages 57% in emergency operations) [3]. To avoid such catastrophic vascular events, essential treatments to progressive aortic dilation is aorta and/or aortic valves replacement.

Although surgical method improved greatly the prognosis of thoracic aortic aneurysm (TAA), uncertainties still remained in decision of time to start surgical intervention, partly because the imaging diagnosis and morphological presentation of TAA are not sufficient to give strong evidences for the timing of surgeries. According to a large clinical study in 2007, 59% of Type A aortic dissections occurred in patients with an aortic diameter of less than 5.5 cm, and 40% occurred in patients with an aortic diameter of less than 5.0 cm [4]. Furthermore, the adjacent distal aorta still has a potential risk of dilation after a successful aortic replacement surgery [5], resulting in reoperations that poses extra risk on the patients.

Recent studies reveal complexing and unique pathogenesis about BAV-related TAA including gene mutations [6], hemodynamics, mechanical stress [7], oxidation and inflammation, and their interactions. As a research hotspot up-to-date, miRNA has attracted much attention in regulating gene expression through translation repression and messenger RNA (mRNA) decay, which enables the abnormal expression of miRNA to participate in the occurrence and progression of various human diseases [8]. Especially, miRNA can be an important regulatory molecule in the studies of some diseases with high heterogeneity and unclear pathogenesis.

In this review, we try to summarize functions of miRNA and relationships between miRNA and BAV-related TAA; and to discuss potential applications of miRNA in diagnosis and treatment for BAV-related TAA.

## Biological function of miRNA

miRNAs are a series of endogenous expression non-coding RNAs (ncRNA) that have a sequence-specific model guiding to the targets. They are classified into different families according to their seed sequences which are the main fragments binding to target genes [9]. Pre-miRNA is a semi-mature molecule with double strands to be separated. One strand is degraded to maintain the homeostasis of functional miRNA, the other strand functions actively to bind to specific regulating targets, including protein-coding mRNA and ncRNA [10].

miRNA cooperates with Argonaute (AGO) proteins to work as a functional complex [11]. AGO2 is ubiquitously intracellular expressed and participate in various of functions [12]. Domains in AGO are involved in the process of miRNA seed sequences recognizing target sites, and this process of recognition and binding is not highly conserved. In addition to strongest complementarity, there are other complementary ways with weak seed-match degrees [8]. The binding targets locate mainly within the 3’ untranslated regions (UTR) of mRNA, inducing translation inhibition and promotion of mRNA decay as main effects. Translation inhibition is achieved by assembling initiation factors and mRNA [13], and mRNA decay (major effects of miRNA) is conducted by enzymatic degradation followed by deadenylation of mRNA [14].

As a member of RNA, miRNA can be regulated by transcriptional promoting or inhibiting. What' more, degradation rates and epigenetic repression can also influence miRNAs' content. Post-transcriptional modifications of miRNA, such as uridylation, adenylated and oxidation [15–17], can change the stability and functions of miRNA.

The functions and characteristics of miRNA described above support the involvement of miRNA in the highly heterogeneous pathogenesis of BAV-related TAA.

In addition, most miRNAs have an average half-life of 119 h or slightly longer, but some also have a rapid change in their content [18]; moreover, miRNA remains highly stable in formalin-fixed tissues [19], and this characteristic can also be found in the serum. These specificities of miRNA support it as an effective and reliable biomarker.

## Pathogenesis and phenotypic heterogeneity of BAV-related TAA

### The pathogenic mechanism of BAV-related TAA

The pathogenesis of BAV has not been fully understood. Aortic root aneurysm and ascending aortic aneurysm are seen early in children with BAV, suggesting that genetic factors may be involved in the progression of the BAV-related TAA [20]. Family studies in monozygotic twins and first-degree relatives had revealed correlations between BAV and heredity [21, 22]. Some researchers have identified several risk factors for BAV-related TAA. In some research, mutation of the NOTCH1 gene is most closely related to the pathogenesis of BAV [23], but the positive detection rate of this mutation in isolated cases is not very high [24]. Other gene mutations, such as ROBO4 and MYH6 were also found in BAV patients [25, 26], some even contributed to syndromic phenotypes as well. Genetic factors of BAV-related TAA seem to be diverse, and are probably not quite deterministic or uniquely deterministic.

The influence of hemodynamic factors on the progression of BAV-related TAA has been widely demonstrated in recent years [7, 27]. Abnormal aortic valves structures produce faster blood spurts, resulting in a higher shear stress toward thoracic aortic wall than normal tricuspid valves did [28]. Besides, the increased wall shear stress has an uneven distribution in different aortic locations. With the structural changes of ascending aorta wall (such as atherosclerotic plaque), abnormal blood flow kinetic energy will be converted into static pressure, a continuous effect on the aorta [29]. Hemodynamic abnormalities weaken the structure of the aorta through the direct mechanical effect, leading to dissection or rupture, such abnormalities also affect the physiological activities of various cells in the aorta, including miRNAs’ expression [30].

Each pathogenic factor influences phenotypes of BAV-related TAA differently [31]. Those phenotypes can be divided basically into aortic root aneurysm and tubular aortic aneurysm. Aortic root dilation is strongly gene-driven, as to tubular phenotype, more hemodynamic inclined [32].

Since no single theory can fully explain its pathogenesis, BAV-related TAA may be a multifactorial disease. As the two important pathogenic factors mentioned previously, miRNA is involved in the post-transcriptional regulation of genes or act as hemodynamic-sensitive molecules.

### The phenotypes of BAV malformation and BAV-related TAA

Heterogeneous as BAV is, classification of the valve malformation patterns and generalization of the thoracic aorta distension types are necessary.

**Fig. 1:**
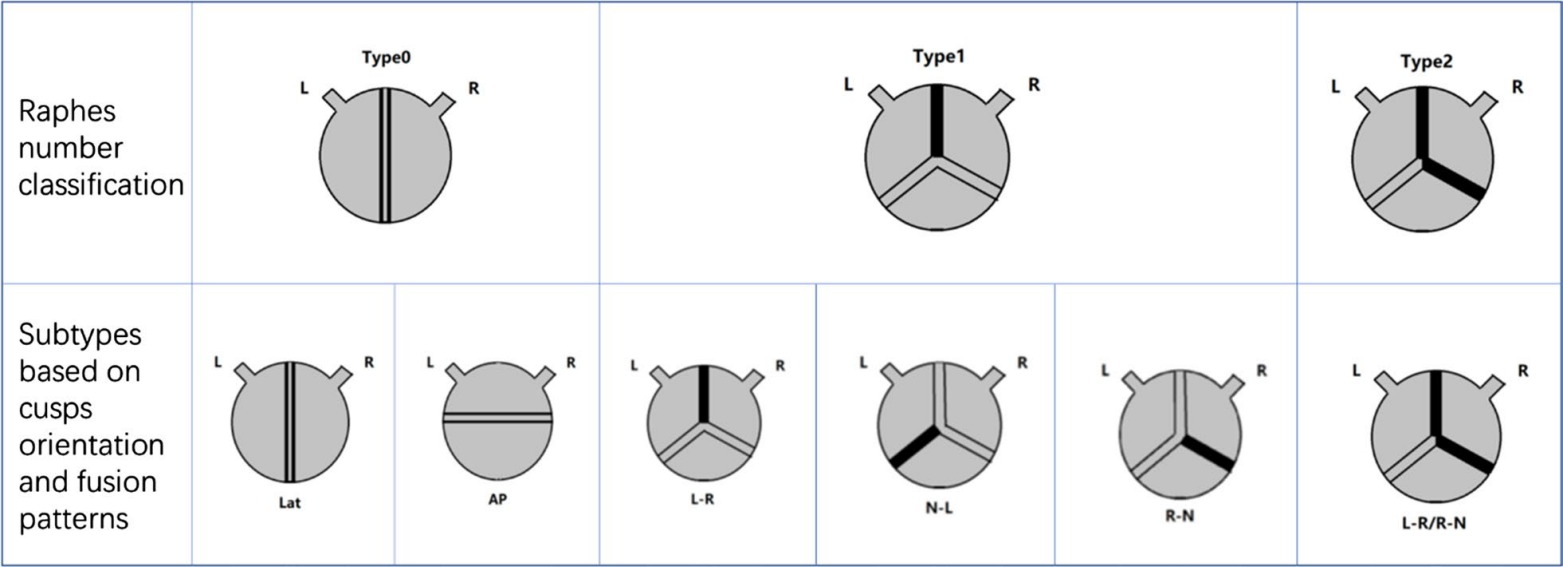
Type 0 has no raphe (left), the direction of transverse or longitudinal openings is used as the basis for further classification. Type 1 has three subtypes (middle), of which L-R fusion has the highest incidence in patients with BAV malformation. Type 2 has bi-commissural fusions (right)

Currently, BAV malformations are mainly detected by echocardiography and classified by Sievers classification, which is shown in Fig. 1 [33]. In a multicenter study involving patients, 1881 out of 2118 patients with BAV had fusion raphes (88.8%), and the remaining 11.2% were Type-0. The phenotype with the highest incidence was the fusion of the left and right coronary cusps (1435 patients, accounting for 76.3%), followed by the right-noncoronary cusps fusion (343 patients, accounting for 18.2%) [34].

According to the location of dilated aortic segment, TAA is mainly divided into root dilation and tubular aorta dilation, and these two phenotypes account for 96% of patients with BAV-related TAA. The remaining 4% patients exhibit dilation in distal part of the ascending aorta, including proximal aortic arch. Tubular aorta dilation is of highest incidence, followed by root dilation, with less distal aorta dilation [35].

In a study by Della Corte A et al., two most prevalent patterns of BAV malformations are correlated with two different dilated segments of aorta: the fusion of the left and right coronary cusps at the root dilation, and the fusion of the right and noncoronary cusps at the tubular dilation. These relationships are morphological only, no correlations were seen between dilated segments and functional integrity of aortic valves [36]. Several studies have examined the relationship between the presence of raphes and the incidence of aortic stenosis and regurgitation in patients with BAV, indicating presence of raphes contributed to increased frequencies of valve dysfunction and greater valvular surgery demands.

Considering the diversity of BAV-related TAA phenotypes, once a study of BAV-related TAA did not differentiate these types, research bias could not be avoided from confounding factors. However, if the study population is not large enough for classification with strict criteria, reliability of the results needs to be discussed. Due to the specificity of root phenotypes, we suggest that root dilatation and tubular dilatation should be sorted broadly.

## Role of miRNA in the pathogenesis of BAV

### Disturbance of the extracellular matrix in the aorta

Extracellular matrix (ECM) is a tissue component mainly composed of collagen, elastin, proteoglycan and aminoglycan. It serves basically as supporting structure of cells, and participant in the cell phenotype regulations through signal transmission [37].

Recent studies on BAV-related TAA have focused on the degeneration of ECM caused by disturbance of matrix metalloproteinase (MMP) / tissue inhibitor of matrix metalloproteinases (TIMP) balance. In 2005, LeMaire SA et al. collected 29 samples of aneurysms (14 BAV-related TAA and 15 TAV-related TAA), and compared them with 14 healthy aorta samples. Differences were found in expression of MMP-2, MMP-9, TIMP-1 and TIMP-9 among the three groups [38]. In 2016, Jie Wu et al. divided BAV-related TAA into mild dilation group and severe dilation group. In aortic samples from the mild dilation group, they found that levels of miR-17 and miRNAs with same seed sequence as miR-17 increased significantly; while TIMP-1, TIMP-2 and TIMP-3 levels decreased and MMP-2 activity increased. In vitro experiments confirmed that miR-17 directly targeted TIMP-1 and TIMP-2 [39]. This study provided some evidences accounting for the continuous progression of BAV-related TAA.

In a bioinformatics study, a miRNAs cluster (located in the 14q32 locus on chromosome 14) centered on miR-494 was screened, and functional enrichment analysis of the cluster revealed that ECM was an important regulatory target of this cluster [40]. In addition, some studies have focused on the correlation between the expression of miRNAs with MMP/TIMP. In 2020, research of Shiho Naito et al. found that in BAV-related TAA aorta tissues, expression of miR-133a was positively correlated with TIMP-1 and 2, and expression of miR-143a was negatively correlated with MMP-2 [41]. Although the explorations of downstream cell signals and the potential targets of miRNA were not involved in these studies, the correlations also support the potential co-role of miRNA and MMP/TIMP in BAV-related TAA.

### Regulation on the phenotype of aortic smooth muscle cells

Vascular smooth muscle cells (VSMCs) are mainly located in the middle thoracic aorta. Conversion from contractile phenotypes to secretory ones, abnormal proliferation and apoptosis of VSCMs can affect the vascular structure [42]. Some studies have focused on how miRNA contributed to BAV-related TAA progression by influencing VSMC phenotype. A study in 2016 by Sebastian Albinsson et al. showed differential expression characteristics of miRNAs found in different groups (BAV, TAV and heart transplant control groups) and different regions (convex and concave aortic regions). They further found that increase of miR-146-5p and miR-21-5p, which regulate the phenotype of SMC synthesis, and decrease of miR-133A-3p, which regulate the phenotype of SMC contraction, could be detected significantly in the convex region of BAV-related TAA [43]. In 2017, next-generation sequencing found that, compared with TAV-related TAA group, 12 miRNAs (2 up-regulated and 10 down-regulated) were differentially expressed in the BAV-related TAA group, among which miR-424-3p and miR-3688-3p were significantly down-regulated. These miRNAs were mainly involved in Hippo signaling pathway, ErbB signaling pathway and TGF-β signaling pathway. Coincidentally, these pathways are associated with cell proliferation and apoptosis. Although no evidences reported to support linkages between these signaling pathways and specific cell types (e.g., VSMC), these findings might lead to new ways for further researches [44].

Aortic dissection is one of the serious complications of BAV-related TAA, and miRNA also influences VSMC in dissection diseases, especially regulates apoptosis of VSMC. miRNA-related apoptosis in aortic dissection was initially identified in abdominal aortic aneurysms by Maegdefessel et al. [45], showing that miR-21 regulated VSMC apoptosis in murine models. In the cases of thoracic aorta dissection, researchers have found that miR-320d and miR-582 decrease in the thoracic aortic dissection tissues, and these two miRNAs were proved to be related to apoptosis by targeting the 3'UTR region of TRIAP1 and NET1 [46]. However, these studies on thoracic aortic dissection did not include BAV malformation, expression characteristics of miRNA and the relationship between miRNA and VSMC apoptosis may be different under BAV-related background.

### Endothelial injury and endothelial phenotypic changes

Recent studies have shown that endothelial cells (ECs) are damaged by abnormal aortic blood flow, and phenotypic changes of ECs have been found in BAV-related TAA aortic tissues. These changes tend to participate in the weakening of the aortic structure of TAA, and may be inducers for aortic rupture or dissection. In a 2017 study, elevated PECAM + endothelial microparticles, shaping from the plasma membrane of ECs during activation, injury, or apoptosis, were found to be associated with aortic dilation in patients with BAV; moreover, a miR-494-centered miRNAs network was found associating with PECAM + endothelial microparticles [40]. According to study of Maleki et al. [47], the miR-200 family participates in the pathogenesis of BAV-related TAA through ECs. Compared with TAV group, expression of miR-200 family (miR-200c and miR-429) decreased in patients with BAV, while expression of their target genes increased. In vitro experiments also supported the involvement of miR-200 in the process of endothelial/epithelial mesenchymal transition (EndMT/EMT) [47].

These studies suggested the important role of miRNA-regulated ECs in the chronic structural weakness and acute structural destruction of BAV-related TAA.

### Oxidative stress intolerance and calcification

In addition to ECM, VSMC phenotype and EC phenotype, intolerance to oxidative stress was also mentioned in some studies. In a study by Julie A et al. which focused on metallothionein (an antioxidant that is upregulated in response to oxidative stress), compared with the control group, 110 genes from patients with BAV-related TAA were changed, and 8 out of which were metallothionein-related. This indicated that BAV aorta was more sensitive to oxidative stress, BAV aortic dilation risk increased under oxidative stress [48]. Recently, abnormal regulation of miR-328-3p in the aortic valve of patients with BAV was reported to induce oxidative stress intolerance in valvular ECs [49]. Although no explicit associations of oxidative stress with miRNA-mediated BAV-related TAA were identified, these experiments provided part of evidences for pathogenesis of BAV-TAA through the regulation of oxidative stress by miRNA.

Calcification is one of the common pathological changes of aortic valves. miR-195 is down-regulated in patients with BAV and valve calcification was promoted through SMAD7 pathway [50]. miR-330-3p contributed to BAV aortic valve calcification by targeting CREBBP [51]. These studies also showed greater hemodynamics changes under the condition of valve calcification, proposed possibilities of structural weakness of aorta itself due to calcification.

### Regulation of miRNAs

There are two ways of regulation on miRNA in the pathogenesis of BAV-related TAA, namely the transcription regulation and the epigenetic modification after transcription.

In the study by Alijbegovic et al., they speculated that actin polymerization promoted VSMC-specific gene expression through transcriptional coactivator MRTF in aneurysm, which may also include transcription regulation of miRNA [52]. As a study mentioned previously, miR-200 family regulates EndMT/EMT process, which suggested a high chromatin occupancy of miR-200c promoters by ZEB1/ZEB2, and a double-negative feedback loop of miR-200c and ZEB1/ZEB2 was more highly activated in patients with BAV [47].

Post-transcriptional modification of miRNA has not yet been studied in BAV-related TAA. However, in a study on cardiac hypertrophy in 2020, a modification of reactive oxygen species (ROS) at site 7 of the seed sequence of miR-1 was mentioned, and oxidized miR-1 played a special role in hypertrophic process of myocardium[17]. Post-transcriptional modification of miRNAs can also be involved in BAV-related TAA by analogy.

## miRNAs as biomarkers for BAV-related TAA

Referring miRNAs as biomarkers, oncology studies will be covered in a lot. Stabilities of miRNAs in formalin-treated tissues [53] and circulating serum [54] enabled them effective biomarkers to predict the lesion progression of the aorta.

Studies on miRNAs as biomarkers of BAV-related TAA has been started long ago, drawing a series of conclusions through different phenotypic grouping methods and different correlation analyses. In 2013, expressions of miR-1 and miR-21 were found to be differed in serum of BAV-related and TAV-related TAA; and miR-133a was associated with the incidence of aneurysm [55]. Martinez-Micaelo et al. analyzed the expression level of miRNAs in the circulation of BAV-related aortic disease and found that miR-122, miR-130a and miR-486 were significantly affected by valve morphology. Moreover, miR-718 was negatively correlated with aortic diameter and could be used as an independent predictor of aortic dilatation, and it was enriched in vascular-remodeling-relating genes [56]. Girdauskas et al. [57] divided the dilated aorta into mild dilated and severe dilated aorta groups. In patients with BAV-related mild dilated aorta, miR-17 and miR-106a expressions were significantly higher than those of patients with severe dilated aorta, and significant reductions of miR-17 and miR-106a were observed in patients with BAV who experienced an adverse aortic event during subsequent follow-ups [57]. Another study in 2018 linking miRNA serum expression with NOTCH1 mutation revealed that miR-145 expression was significantly lower in a subgroup of patients with the NOTCH1 mutation than those without [58]. By A. Gallo et al., miR-15b was found to be an independent predictor of aortic dilatation, and the circulating level of miR-34a was negatively correlated with aortic wall elasticity in patients with BAV [59]. Lately a significant negative linear correlation was found between circulating miR-17, miR-20a, miR-106a and proximal aortic diameter [60].

**Fig. 2:**
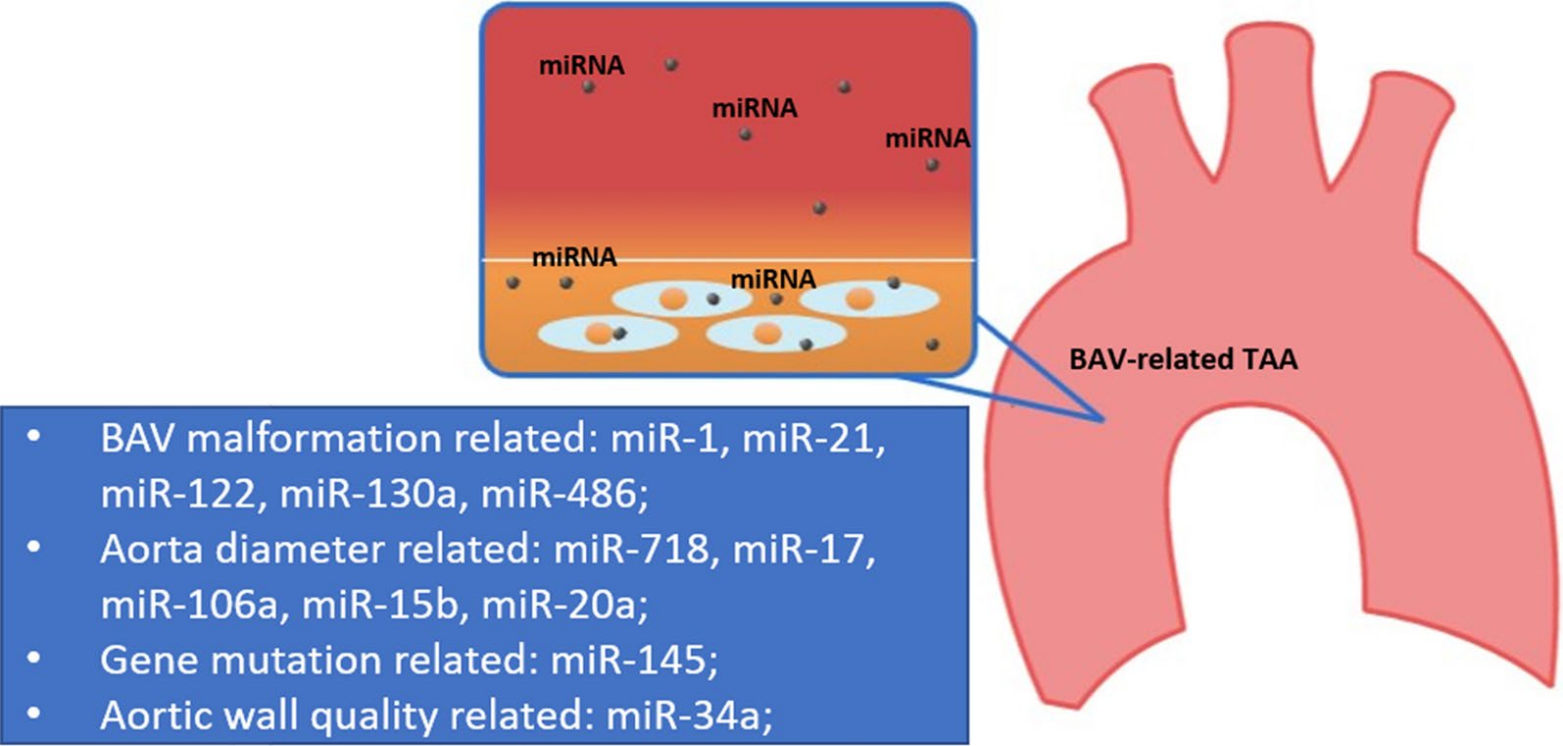
In addition to directly involving in the pathogenesis of BAV-related TAA by affecting extracellular matrix (ECM), vascular smooth muscle cells (VSMCs) and endothelial cells (ECs), miRNAs can also enter the circulatory system as biomarkers

As is shown in Fig. 2, miRNAs are used as circulating biomarkers mainly to identify BAV malformation (miR-1, miR-21, miR-122, miR-130a, miR-486), to confirm dilated diameter of the aorta (miR-718, miR-17, miR-106a, miR-15b, miR-20a), to check gene mutation (miR-145), and to clarify aortic wall characteristics (miR-34a). Imaging means up-to-date, such as echocardiography and computerized tomography (CT), is not accurate enough in determining BAV malformation and aneurysm diameter. miRNA biomarkers carrying more clinical significances could be conducive to illustrate the characteristics of aortic wall and the progression of BAV-related TAA.

Giving complex pathogenic backgrounds about BAV-related TAA, each single biomarker may not be reliable enough in specificity, sensitivity, and biological significance. Setting up a predictive model that includes a series of related biomarkers will be more credible for clinic applications.

## Conclusion

Currently, much difficulties and confusions still lie in the treatment for BAV-related TAA. In this review, we show that miRNA has some perspective to address such problems.

Firstly, current studies have shown that miRNA affects ECM, VSMC and ECs by regulating the downstream pathways epigenetically, providing possible targets to intervene preoperatively and postoperatively to ameliorate aneurysm progression.

In the cell biology studies of BAV-related TAA, we believe that the research-target of miRNA itself and its downstream pathways are noteworthy in the following directions: (A) Post-transcriptional oxidative modification of miRNAs: As mentioned above, the characteristics of oxidative stress intolerance of BAV-related TAA provide objective conditions for oxidative modification. Oxidative modification of miRNA, especially in its seed sequence, will change its targeted binding site. Studies in this direction could help us to more accurately describe the expression characteristics and biological functions of miRNAs in BAV-related TAA. (B) Circular RNA: circular RNA can act as a sponge for miRNA, thus alleviating the inhibition of miRNA on target mRNA [61]. The studies of circular RNA can help us find a more complete regulatory axis of miRNA in the pathogenesis of BAV-related TAA.

Clinical application of miRNAs in BAV-related TAA: In addition to the intervention on miRNA-related downstream pathways and regulatory axis, the study of Yang H et al. in 2019 on miRNA delivery system constructed by nanoparticles for the treatment of myocardial infarction suggested that we could use protective-miRNA delivery system to delay the disease progression of BAV-related TAV [62]. In this part, the selection of protective-miRNA and the study of delivery systems are of great value.

Secondly, miRNAs are potential as biomarkers for this disease due to the tissue/circulating-stable characteristics, and can be used as a supplement for imaging means to determine surgery time and help decision-making in BAV-related TAA. Current studies have not found that a single miRNA has sufficient prediction accuracy for BAV-related TAA, and a prediction model involving multiple miRNAs is worthy of expectation. Moreover, most of the current studies on miRNAs as biomarkers only show statistical correlation, and the evidence of the association between these miRNAs and the pathological progression of BAV-related TAA also needs to be further studied. Although biomarker applications of these miRNAs need to be further validated in large scaled studies, such molecules are promising and we hope they will eventually be helpful in diagnosis and treatment of BAV-related TAA.

## Data Availability

Not applicable.

## Abbreviations

BAV: Bicuspid aortic valve
TAA: Thoracic aortic aneurysm
VSMC: Vascular smooth muscle cells
ECM: Extracellular matrix
EC: Endothelial cell
MMP: Matrix metalloproteinase
TIMP: Tissue inhibitor of matrix metalloproteinases
